# Phylogeny, ancestral ranges and reclassification of sand dollars

**DOI:** 10.1038/s41598-023-36848-0

**Published:** 2023-06-23

**Authors:** Hsin Lee, Kwen-Shen Lee, Chia-Hsin Hsu, Chen-Wei Lee, Ching-En Li, Jia-Kang Wang, Chien‑Chia Tseng, Wei-Jen Chen, Ching-Chang Horng, Colby T. Ford, Andreas Kroh, Omri Bronstein, Hayate Tanaka, Tatsuo Oji, Jih-Pai Lin, Daniel Janies

**Affiliations:** 1 National Museum of Marine Biology and Aquarium, Pingtung, 944401 Taiwan; 2 Department of Geosciences, National Taiwan University, Taipei, 10617 Taiwan; 3 Institute of Oceanography, National Taiwan University, Taipei, 10617 Taiwan; 4 Biology Department, National Museum of Natural Science, Taichung, 40453 Taiwan; 5 Tuple LLC, 2413 Commonwealth Ave, Charlotte, NC 28205 USA; 6 School of Data Science, University of North Carolina at Charlotte, 9201 University City Blvd, Charlotte, NC 28223 USA; 7 Department of Bioinformatics and Genomics, University of North Carolina at Charlotte, 9201 University City Blvd, Charlotte, NC 28223 USA; 8 Center for Computational Intelligence to Predict Health and Environmental Risks (CIPHER), University of North Carolina at Charlotte, 9201 University City Blvd, Charlotte, NC 28223 USA; 9 Department of Geology and Palaeontology, Natural History Museum Vienna, 1010 Vienna, Austria; 10 School of Zoology, Faculty of Life Sciences, Tel Aviv University, 6997801 Tel Aviv, Israel; 11 Steinhardt Museum of Natural History, Tel Aviv University, 6997801 Tel Aviv, Israel; 12 Department of Biological Sciences, Graduate School of Science, University of Tokyo, Tokyo, 113-0033 Japan; 13 University Museum, Nagoya University, Furo-cho, Nagoya, 464-8601 Japan

**Keywords:** Ecology, Evolution, Systems biology, Zoology, Ecology

## Abstract

Classification of the Class Echinoidea is under significant revision in light of emerging molecular phylogenetic evidence. In particular, the sister-group relationships within the superorder Luminacea (Echinoidea: Irregularia) have been considerably updated. However, the placement of many families remains largely unresolved due to a series of incongruent evidence obtained from morphological, paleontological, and genetic data for the majority of extant representatives. In this study, we investigated the phylogenetic relationships of 25 taxa, belonging to eleven luminacean families. We proposed three new superfamilies: Astriclypeoidea, Mellitoidea, and Taiwanasteroidea (including Dendrasteridae, Taiwanasteridae, Scutellidae, and Echinarachniidae), instead of the currently recognized superfamily Scutelloidea Gray, 1825. In light of the new data obtained from ten additional species, the historical biogeography reconstructed shows that the tropical western Pacific and eastern Indian Oceans are the cradle for early sand dollar diversification. Hothouse conditions during the late Cretaceous and early Paleogene were coupled with diversification events of major clades of sand dollars*.* We also demonstrate that Taiwan fauna can play a key role in terms of understanding the major Cenozoic migration and dispersal events in the evolutionary history of Luminacea.

## Introduction

The irregular echinoids of the order “Clypeasteroida” sensu A. Agassiz 1872–1874 or “sand dollars” sensu lato, constitute a morphologically well-defined group of burrowing sea urchins living on sandy bottoms from the intertidal to the deep-sea^1–3^. Because of their characteristic flat body, they are commonly known as sand dollars or sea biscuits and were previously subdivided into two suborders “Scutellina” and “Clypeasterina”, respectively. However, the monophyly of this traditionally defined morpho-group has been challenged since the advent of molecular studies^4–9^. Members of the former suborder “Scutellina” were resolved as sister group of irregular echinoids of the suborders Cassiduloida + Echinolampadoida^7^, and together, they were sister to the “Clypeasterina”^7,8^. Based on molecular evidence, Mongiardino Koch et al.^8^ proposed an elevation of the suborder “Scutellina” to "Scutelloida" and restriction of usage of the order "Clypeasteroid" solely to member of the former suborder "Clypeasterina". To date, four modern irregular echinoid clades, Clypeasteroida, Scutelloida, Cassiduloida, and Echinolampadoida are recognized; these taxa together constitute the clade Luminacea^10^. Luminacea first appeared in the Middle to Late Jurassic^7^. As bioturbators, they provide significant services to the marine ecosystem. Dead Luminacea also contribute an important part of shallow marine sediments (e.g., in form of sand dollar mass deposits^11^) and have indirectly influenced the ecosystem functions responding to global changes, in modern time as well as in the past.

Due to their good fossil record, the Luminacea were considered one of the best examples to document echinoderm spatio-temporal evolution^12^. Originating in the Early Cretaceous^13^, the Cassiduloida and Echinolampadoida were extremely diverse. Their diversity peaked during the Eocene^7,14^, in which 60% of all echinoids found from this period belong to these groups^15^. Since then, the number of living species dramatically decreased. At present, there are 28 extant species and hundreds of fossil species described from the suborders Cassiduloida and Echinolampadoida. Compared to the cassiduloids and echinolampadoids, the diversification of the other luminacean clades: Scutelloida and Clypeasteroida, are relatively recent, with no known fossil record before the Eocene^1,6,13,16^, which is in conflict with molecular phylogenetics based hypothesis, suggesting that the order Clypeasteroida is sister to all other luminacean echinoids. As the fossils of Luminacea exhibit high preservation potential, they can be easily identified and classified at least to order level. The incongruence between paleontological evidence and recent phylogenetic hypothesis based on molecules, highlights the need for further investigation to resolve the early evolution of the Luminacea^5,7,8,13^.

To date, there are 173 extant species of Luminacea^17^, which are globally distributed with considerably more species in the tropical Indo-West Pacific region, especially in southeast Asian waters^18^. Extant species are currently classified under three orders and 14 families. The Cassiduloida comprises three families: Cassidulidae, Eurhodiidae, and Neolampadidae^7^. The Echinolampadoida comprises a single family: Echinolampadidae; while Clypeasteroida includes two families: Arachnoididae and Clypeasteridae, respectively. The Scutelloida includes nine extant families. Based on morphology, the included Fibulariidae and Laganidae can be further grouped into an infraorder Laganiformes whereas Echinarachniidae, Taiwanasteridae, Astriclypeidae, Dendrasteridae, Mellitidae, and Scutellidae are grouped into another infraorder, Scutelliformes^13,15,18–22^. The classification of the remaining family Rotulidae is still unsettled. Some studies placed it within the Laginiformes^15,18,21,22^, whereas others consider it as a member of the Scutelliformes^13,19,20^.

**Figure 1:**
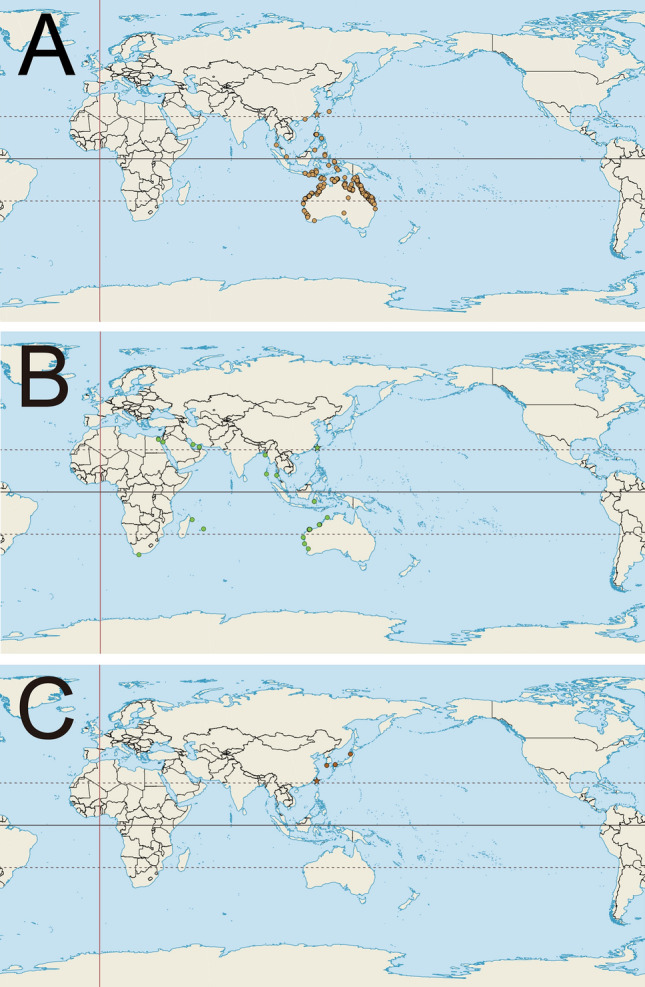
Three types of global distribution patterns based on occurrence data recorded in the Global Biodiversity Information Facility (GBIF) (Table 1). (**A**) Occurrences of *Peronella lesueuri* (L. Agassiz, 1841) showing a longitudinal distribution in the Pacific-West, at both northern and southern hemispheres (GBIF; https://doi.org/10.15468/dl.uftmga; Table 1). (**B**) Occurrences of *Sculpsitechinus auritus* (Leske, 1778) showing an Indo-West-Pacific (IWP) distribution, including the Red Sea and Persian Gulf (GBIF; https://doi.org/10.15468/dl.hbqzud; Table 1). (**C**) Occurrences of *Astriclypeus mannii* Verrill, 1867 showing endemism in the region of Japan, South Korea and Taiwan (GBIF; https://doi.org/10.15468/dl.jdvqfb; Table 1). Maps were created with QGIS (https://qgis.org/, version 3.0.3).

Regarding the distribution of modern Clypeasteroida and Scutelloida, the seas around Taiwan are unique due to mixing of surface ocean currents. The warm, Kuroshio Branch Current coming from the tropical zone and the cold, China Coastal Current coming from the temperate region meet in the Taiwan Strait^23^. Consequently, a mixture of warm- (e.g., *Arachnoides placenta*^24^) and cold-water (e.g., *Scaphechinus mirabilis*^23^ and *Astriclypeus mannii*; Table 1) species occur in Taiwan. Based on the global distributions of sand dollars sensu lato, three biogeographic patterns seem to intersect at this area (Fig. 1A–C). Pattern 1, exemplified by *Peronella lesueuri* (Fig. 1A), which exhibits a longitudinal distribution ranging from the Northwest Pacific to South Australia. Other taxa with similar distributions include *Arachnoides placenta*, *Clypeaster virescens,* and *Fibularia plateia*. Pattern 2, exemplified by *Sculpsitechinus auritus* (Fig. 1B), exhibits a latitudinal distribution, covering the Indo-West Pacific. Other taxa with similar distributions include *Clypeaster reticulatus*, *Laganum fudsiyama* and *Echinocyamus megapetalus*. Pattern 3, exemplified by *Astriclypeus mannii* (Fig. 1C), shows a narrow distribution, restricted to the region of Japan, South Korea and Taiwan. Other taxa in this group include *Sinaechinocyamus mai* and *Scaphechinus mirabilis*. High diversity and disparity of sand dollars sensu lato at both subtidal and intertidal coastal regions, combined with three distinct biogeographic patterns (Fig. 1A–C), indicate that Taiwanese waters could be a major migration juncture for the Luminacea^12,25^.

**Table 1:** Global occurrences of clypeasteroids reported from Taiwan waters.

Faunal provinces	Taxa	Data sources
West Pacific	*Clypeaster virescens* Döderlein, 1885	https://doi.org/10.15468/dl.7i7hje
	*Arachnoides placent*a (Linnaeus, 1758)	https://doi.org/10.15468/dl.wswvtk
	*Peronella lesueuri* (L. Agassiz, 1841)	https://doi.org/10.15468/dl.uftmga
	*Fibularia plateia* H.L. Clark, 1928	https://www.gbif.org/occurrence/download/0005913-190621201848488
Indo-West Pacific	*Sculpsitechinus auritus* (Leske, 1778)	https://doi.org/10.15468/dl.hbqzud
	*Clypeaster reticulatus* (Linnaeus, 1758)	https://www.gbif.org/occurrence/download/0005918-190621201848488
	*Laganum fudsiyama* Döderlein, 1885	https://www.gbif.org/occurrence/download/0005919-190621201848488
	*Echinocyamus megapetalus* H.L. Clark, 1914	https://www.gbif.org/occurrence/download/0005914-190621201848488
Endemic to East Asia	*Astriclypeus mannii* Verrill, 1867	https://doi.org/10.15468/dl.jdvqfb
	*Sinaechinocyamus mai* (Wang, 1984)	Refs^26–28^
	*Scaphechinus mirabilis* A. Agassiz, 1864	https://doi.org/10.15468/dl.yfwncs

Luminacea includes irregular echinoids with spectacular diversification of mobile marine faunas during the Mesozoic Marine Revolution that now constitutes one of the most important components of echinoid fauna in modern seas^29^. This superorder, however, is in need of taxonomic revision, because its classification (see Electronic Supplementary Material 1) is not fully resolved yet^7^. In this study, we inferred the phylogeny of extant Luminacea based on a multi-gene dataset with denser taxon-sampling in Clypeasteroida and Scutelloida compared to previous DNA-based studies. A time-calibrated phylogenetic tree based on a relaxed Bayesian molecular clock and eight robust fossil calibration points was reconstructed to provide a timescale for the origin and diversification of the Luminacea and its main lineages. This time tree was then used as a framework to test existing hypotheses regarding the biogeographical history of the Luminacea.

## Results

### Phylogenetic reconstruction

**Figure 2:**
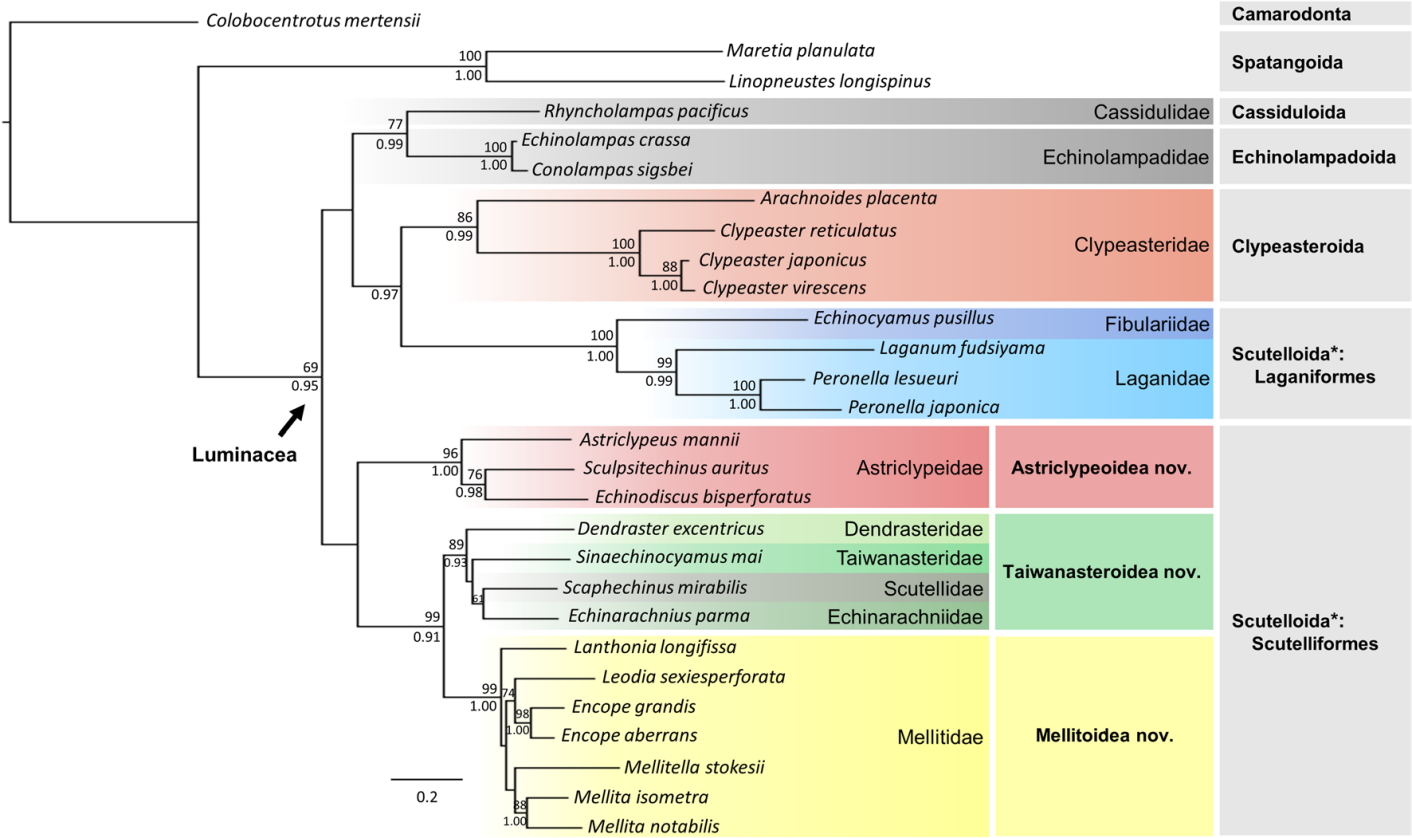
Phylogenetic relationships of the Luminacea inferred using partitioned Maximum Likelihood analysis based on 3301 bp long concatenated multi-gene sequences. Asterisk represents polyphyletic Scutelloida. Nodal supports are shown as bootstrap (BS) values in percentage (above) and posterior probabilities (PP) (below). Values below 60% in BS and 0.8 in PP are not shown. Colored rectangles highlight the resolved main clades. Figure was made with Microsoft PowerPoint (https://www.microsoft.com/, version 2016).

We reconstructed the phylogeny of the Luminacea based on a combined dataset containing DNA sequences from both mitochondrial (*cox1* and *16S*) and nuclear (*28S* and *H3*) gene fragments from 28 echinoderm taxa. The topologies of inferred Maximum Likelihood (ML) and Bayesian Inference (BI) trees (Fig. 2 and Fig. S1, respectively) were largely identical. Within the Luminacea, five well- to moderately-supported clades were resolved: Cassiduloida + Echinolampadoida clade, Clypeasteroida clade, Laganiformes clade, the Astriclypeidae clade, and the clade containing Dendrasteridae, Echinarachniidae, Mellitidae, Scutellidae, and Taiwanasteridae. These phylogenetic results confirmed the monophyly of the Cassiduloida + Echinolampadoida and Clypeasteroida (sensu Mongiardino Koch et al.^8^, ^7^) yet rejected that of Scutelloida. The Scutelloida is composed of two distinct clades with one clade (Laganiformes) resolving as sister to the Clypeasteroida and the other (Scutelliformes) as sister to the clade containing the Cassiduloida, Echinolampadoida, Clypeasteroida and Laganiformes in our analysis. In accordance with the results of Mongiardino Koch et al.^10^, within the Scutelliformes, the families Dendrasteridae, Taiwanasteridae, Scutellidae, and Echinarachniidae formed a monophyletic group, which was sister to the Mellitidae (Fig. 2).

### Time-calibrated phylogenetic tree

**Figure 3:**
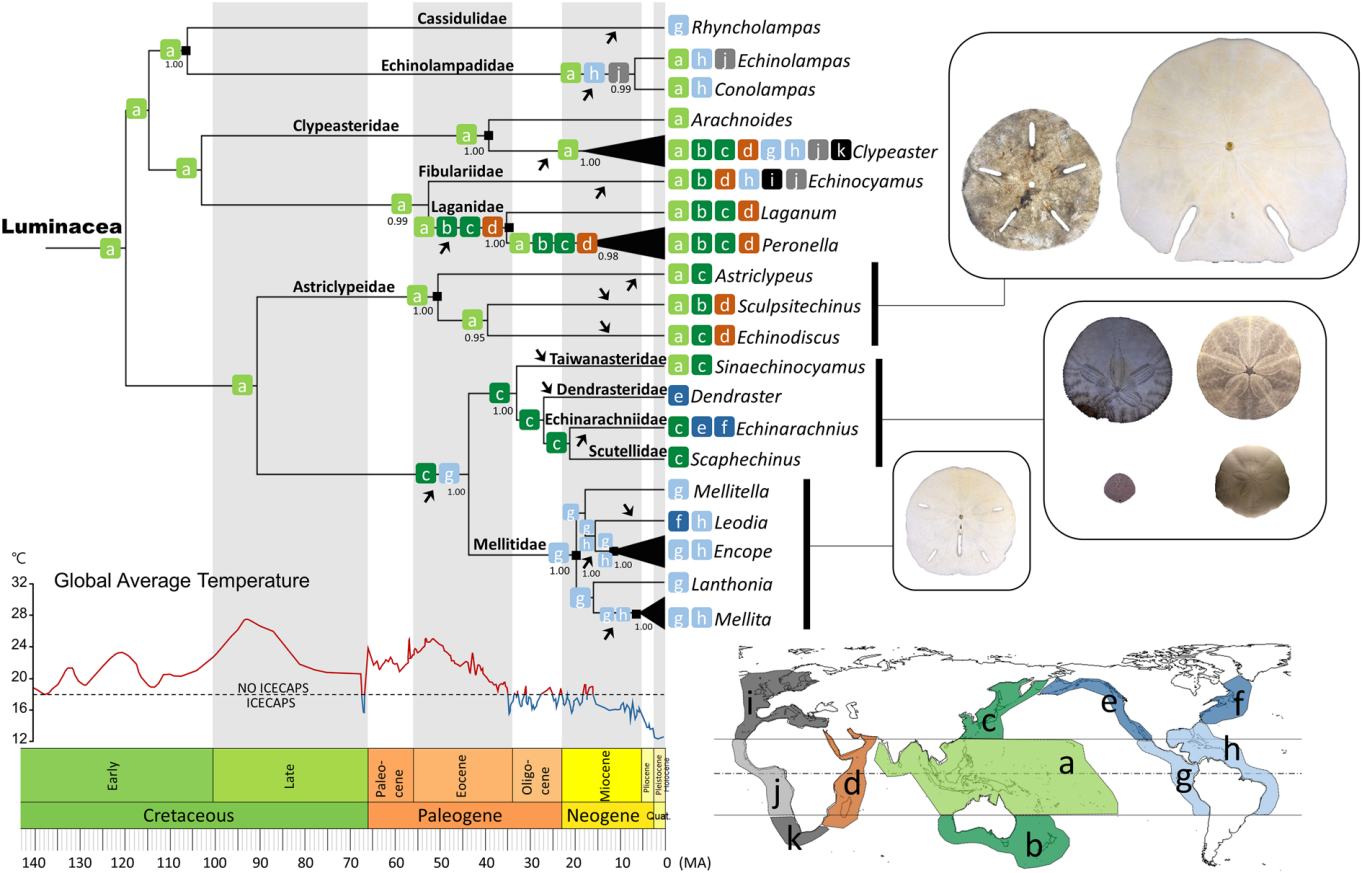
Most-likely ancestral range reconstruction of the Luminacea using the Dispersal–Extinction–Cladogenesis (DEC) model on the simplified Bayesian phylogenetic tree inferred by BEAST v.2.6.7^30^ based on *cox1*, *16S*, *28S*, and *H3* data. Outgroups were omitted from this analysis. Nodes represent the median divergence times. Values near nodes represent posterior probabilities (PP). PP values below 0.95 are not shown. Shapes of lunule from the corresponding scutelloid clades are shown to the right. Colored, lettered boxes on the nodes represent the most likely areas of origin (lower right; **a.** Tropical eastern Indian Ocean and western Pacific Ocean (EIWP); **b.** Southern Australia and New Zealand (SANZ); **c.** Northwestern Pacific (NWP); **d.** Tropical western Indian Ocean (WIO); **e.** Northeastern Pacific (NEP); **f.** Northwestern Atlantic (NWA); **g.** Tropical eastern Pacific (EP); **h.** Tropical western Atlantic and Caribbean Sea (WA); **i.** Northeastern Atlantic and Mediterranean (NEA); **j.** Tropical East Atlantic (EA); **k.** South Africa (SAFR)) reconstructed by RASP v.4.2^31^. Filled squares represent the constrained and assigned age prior nodes. Black arrows indicate inferred events of range expansions. Graph of Phanerozoic paleotemperatures was modified from Scotese et al.^32^. Figure was made with Microsoft PowerPoint (https://www.microsoft.com/, version 2016). Image credit: Jih-Pai Lin.

A BI time-calibrated phylogenetic tree based on a concatenated gene sequence dataset (*cox1*,*16S*, *28S* and *H3*) was reconstructed using a relaxed log-normal clock model with eight well-documented fossil calibration points (see Table S1). The resulting topology was nearly identical to the ML and BI tree (Figs. 2 and 3) except a few shallow branching nodes with weak or without supports (e.g., within the Mellitidae). The divergent time to the most recent common ancestor (MRCA) of the crown group of Luminacea is estimated to be 121.05 million years ago (Ma) with a 95% highest posterior density (95% HPD) of 107.69–136.61 Ma. The origin of modern Cassiduloida + Echinolampadoida is estimated to be 108.72 Ma (102.5–123.79 Ma). The divergent time to the MRCA of the Clypeasteroida and Laganiformes is 111.11 Ma (74.89–166.83 Ma), while that of the Scutelliformes is 91.09 Ma (66.06–112.32 Ma). Although these estimations were older than their oldest fossil records (Eocene) (Fig. 3), they are closer to their oldest known fossil records than those ones estimated in previous studies^7,13^.

### Ancestral area reconstruction

Ancestral area reconstruction based on the time-calibrated tree using the Dispersal–Extinction–Cladogenesis (DEC) model implementing in RASP ver. 4.2^31^ suggests that the Luminacea most likely originated and diversified in the tropical eastern Indian Ocean and western Pacific (EIWP) region during the early Cretaceous before undergoing multiple expansions to other areas (Fig. 3). The ancestral Laganidae (Scutelloida: Laganiformes) were likely widespread in the entire Indo-West Pacific, south of southern Australia and New Zealand, and north to the northwestern Pacific during the Eocene. The common ancestor of the clade containing Taiwanasteridae, Dendrasteridae, Echinarachniidae, Scutellidae, and Mellitidae was likely distributed to the northwestern Pacific and tropical eastern Pacific before the Eocene. Wide-spread genera such as *Clypeaster*, *Echinocyamus*, *Echinodiscus*, and *Echinarachnius* likely underwent multiple range expansions either from the area of origin or by secondary migrations. The broadest distributed genus *Clypeaster* may have had its range expansion by the earliest Miocene (Fig. 3). The full DEC analysis result is shown in Electronic Supplementary Material 1.

## Discussion

Based on the most dense taxon sampling to date, comprising nine out of ten families and 18 out of 29 extant genera of sand dollars sensu lato, our phylogenetic results reject the sister-group relationship between Clypeasteroida and Scutelloida, which is congruent with previous DNA-based studies^4,5,7,8^. The monophyly of the currently used superfamily Scutelloidea Gray, 1825^17^ (including the families Astriclypeidae, Dendrasteridae, Mellitidae, Scutellidae) as well as the Scutelloida^7,8,10^ in other usage should be re-examined. Among the Scutelloida, the sister-group relationship between Laganiformes and Scutelliformes was often suggested by previous studies, yet the phylogenetic inferences were either based on fewer representative genera^4,5,7,8^ or unbalanced character-sampling of a molecular vs. morphology data in combined analyses^7,13^. This relationship is rejected by the present study. We argue that the attributed morphological similarity in these two groups could be the result of convergent evolution.

The phylogenetic position of the family Taiwanasteridae, represented by *Sinaechinocyamus mai*, has been reviewed recently^9,13,21,33,34^. It is closely related to *Dendraster*, *Echinarachnius,* and *Scaphechinus* (Fig. 2). Among them, the eccentric apical disc is an adaptation to the feeding strategy of *Dendraster* and its feeding posture^35^. Other than that, the four groups are similar—non-lunulate, with similar petals (Figs. S3, S4). *Sinaechinocyamus* is a relatively long ranging genus with good fossil records since the late Miocene (~ 8 Ma) in Taiwan^36^. Furthermore, new occurrences of *Sinaechinocyamus* have been found in coastal regions in Japan (Fig. S5), extending the geographic distribution outside of Taiwan Strait.

The new superfamily Taiwanasteroidea is proposed here to include families of Dendrasteridae, Echinarachniidae, Scutellidae, and Taiwanasteridae. Descriptions of Taiwanasteroidea together with other two new superfamilies Astriclypeoidea and Mellitoidea are given at the end of this section.

With the new phylogeny of the Luminacea reconstructed, we demonstrate important evidence on solving the mystery of lunule origins. Seilacher^25^ stated that lunule evolved at least six times in sand dollars. Combining molecular and fossil evidence, Mongiardino Koch and Thompson^7^ proposed a lunulate clade consisting of *Astriclypeus* and *Mellita* (as well as other fossil forms). In our study, lunule belong to two distinct clades (Astriclypeoidea and Mellitoidea) separated by the non-lunulate clade (Taiwanasteroidea) (Figs. 2 and 3). This indicates independent origins for the lunule in the Luminacea. The formation of lunule in Mellitidae consists of plates with a festooned arrangement and that is significantly different from the ones in Astriclypeidae with a cross-linked arrangement (see ref.^25^). In a broader sense, the macroevolutionary trend of Echinoidea suggested on the basis of molecular evidence^9^ similarly reflects the “Dollo’s law of irreversibility”^37^, a hypothetical scenario showing: (A) innovation of lantern in early echinoids during the late Paleozoic; (B) loss of lantern at adult stage among early irregular echinoids during the Mesozoic; and (C) independent re-innovation of the lantern with modifications^33^ in adult clypeasteroids and scutelliformes during the Cenozoic.

**Figure 4:**
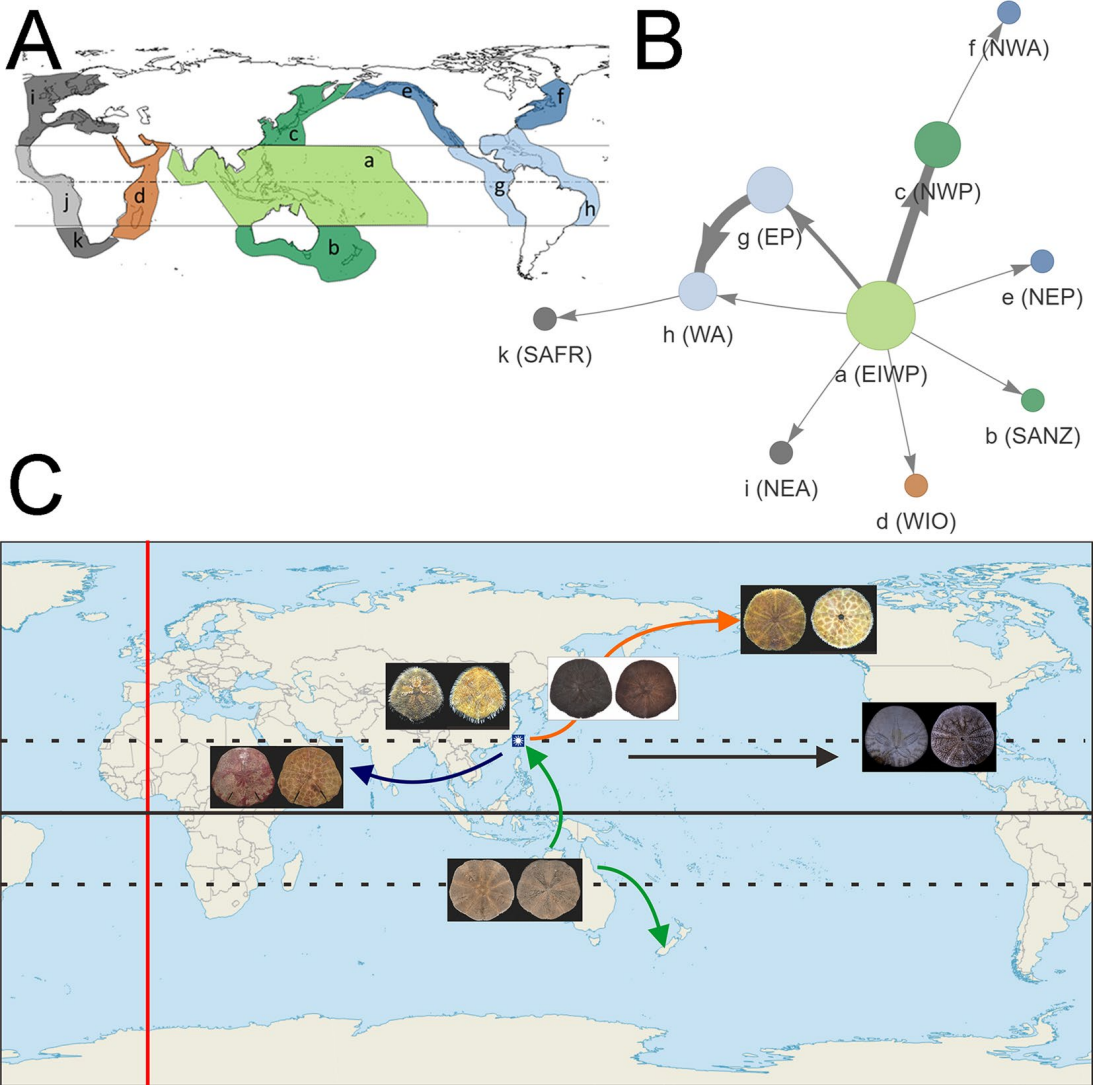
Biogeography and migration network of Taiwanese fauna. (**A**) 11 defined biogeographical regions for Luminacea modified from previous studies^15,18^. **a.** Tropical eastern Indian Ocean and western Pacific Ocean (EIWP); **b.** Southern Australia and New Zealand (SANZ); **c.** Northwestern Pacific (NWP); **d.** Tropical western Indian Ocean (WIO); **e.** Northeastern Pacific (NEP); **f.** Northwestern Atlantic (NWA); **g.** Tropical eastern Pacific (EP); **h.** Tropical western Atlantic and Caribbean Sea (WA); **i.** Northeastern Atlantic and Mediterranean (NEA); **j.** Tropical East Atlantic (EA); **k.** South Africa (SAFR). Figure was made with Microsoft PowerPoint (https://www.microsoft.com/, version 2016). (**B**) StrainHub^39^ biogeographic network. Connections between geographic provinces is indicated by edges. Arrows indicate direction of migration and thickness of nodes indicates frequency. We used the RASP tree (Fig. S1) and geographic metadata to create the network and calculate the source/hub ratio (SHR). The SHR indicates the relative importance of geographic provinces as sources. Larger circles are more important sources of lineages. Figure was generated with StrainHub v0.2.3 on R v4.1.2. (https://doi.org/10.1093/bioinformatics/btz646). (**C**) Hypothesized migration trends for key sand dollar species reported in Taiwan waters. Map was created with QGIS (https://qgis.org/, version 3.0.3).

With new biogeographic analyses presented here (Fig. 4A–C), hypotheses^12,18,25,38^ on the origin(s) of the modern groups of sand dollars can be tested. Smith^12^ suggested that modern sand dollars have three biogeographical radiation hotspots to explain their present-day pattern of diversification. First, the diverse Arachnoidinae (including the genera *Arachnoides* and *Fellaster*) within the Clypeasteroida originated in the Australian region. Second, the ancestral Scutelloida emerged in the Caribbean Sea/ Mediterranean Sea. Third, the ancestral Rotulidae arose in tropical West Africa. Based on the abundant, solid fossil evidence, Seilacher^38^ further noted that Taiwan could be another radiation hotspot where modern Astriclypeidae emerged.

There are 11 geographic provinces defined in this study (Fig. 4A). As such, migration can be well illustrated with the biogeography network visualization software StrainHub^39^. The tropical eastern Indian and western Pacific Ocean (EIWP) have the highest source/hub ratio (SHR). Tropical eastern Pacific (EP) and northwestern Pacific (NWP), and tropical western Atlantic and Caribbean Sea (WA) have moderate SHR (Fig. 4B). Other nodes have low SHR. In addition, we reconstruct the polarity and frequency of migration events between geographic provinces. EIWP to NWP and EP to WA have a high frequency of migration. EIWP to EP has moderate frequency of migration. The other edges show a low frequency of migration.

Results show that EIWP is one of the key biodiversity and migration centers for shallow marine echinoids (Fig. 4B). In addition to Australia that has been identified previously^12,25^, the region around Taiwan is an important migration juncture for key echinoids mentioned here (Fig. 4C). Smith^12^ hypothesized that the clade of Astriclypeidae originated in the Mediterranean Sea based on their common presence in the fossil record of the region. Based on our analyses (Figs. 3 and 4B), however, the clade of modern Astriclypeidae appears to have a reversed migration trend, originating from Asia and then spreading to the Red Sea (Fig. 4C).

The MRCA of Taiwanasteroidea likely started to expand its range from tropical origins to high latitudes along the Pacific coast with warming ocean currents during hothouse conditions. This superfamily gradually gave rise to a cold-water lineage following their range expansion to the northeastern Pacific and northwestern Atlantic during subsequent periods of cooling. A similar model of migration can also be observed in other invertebrates. The range expansions of gastropod superfamilies Turritelloidea and Buccinoidea in the northern Pacific have mainly been influenced by thermal deterioration^40^. The expansions of boreal Buccinidae, Beringiinae, and Turritellinae were caused by the progressive cooling beginning in the Late Eocene^40^. In the genus *Littorina* (Gastropoda: Littorinidae), speciation occurred in response to climatic cooling during the Cenozoic at higher latitudes on the Asian coast^41^. It should be noted that Ghiold and Hoffman^18^ mentioned that the living *Echinarachnius parma*, currently inhabiting the northwestern Atlantic, likely migrated from the Northeast Pacific through the Arctic during the Late Pliocene climatic amelioration (Fig. 3). Extant *Sinaechinocyamus*, on the other hand, subsequently expanded to tropical area and is nowadays abundant in Taiwan.

The inferred historical biogeography of the Luminacea suggests that the common ancestor of this group originated from the EIWP in the early Cretaceous (Fig. 3). The Cassiduloida and Echinolampadoida, appear to have dominated the world’s echinoid diversity, with 60% of all Eocene echinoids belonging to these groups^42^. Since then, the number of species decreased dramatically^7,14^. This implies that the evolution of cassiduloids and echinolampadoids following the Eocene was driven by a series o extinction events. The fossil record thus is fundamental to fully understand their evolutionary histories. Conversely, with dense taxon sampling of extant sand dollars sensu lato, their evolutionary histories are well illustrated.

Echinoids in general, particularly taxa inhabiting shallow subtidal to intertidal habitats, are sensitive to oscillating ocean temperatures^12,43^. The sand dollar sensu lato likely expanded from the origin tropical EIWP to adjacent areas between the late Cretaceous and Eocene, and before the Late Eocene–Oligocene Cooling. Shallow marine currents that regulate the heat flow and governs sea surface temperature is largely driven by wind directions in the troposphere and climate patterns on Earth^44,45^. The relatively higher sea temperatures and warm surface currents^46^ during the late Cretaceous, Paleocene, and Eocene (Fig. 3) drove the dispersal of sand dollars from low- to high-latitudes. Two range expansions likely occurred during this period: the ancestral Laganidae expanded to the south (southern Australia and New Zealand), north (northwest Pacific), and west (tropical western Indian Ocean); and the MRCA of Taiwanasteroidea expanded to the northern Pacific realm (Fig. 3). Vicariance and secondary dispersals likely occurred at various regions as the climate shifted from hothouse to ice-age conditions after the Paleocene–Eocene Thermal Maximum (PETM)^47^ (Fig. 3). For example, the *Dendraster*, *Echinarachnius*, and *Scaphechinus* seem to have adapted to cold-water and survived through modern northern Pacific and northwestern Atlantic; while the Mellitidae is currently the only group that inhabits tropical America^48,49^. The widely distributed genus, *Clypeaster*, likely originated in the western Tethys in the Eocene and has probably undergone several range expansions during the Miocene and spread worldwide.

## Systematics

### Infraorder Scutelliformes Haeckel, 1896

Diagnosis: One lantern support (auricle) on internal side of single interambulacral plate near peristome; interambulacral column ends with two plates at the apical disc; with lunules and/or noticeable marginal indentations; periproct is on oral side and near peristome.

#### Superfamily Astriclypeoidea Lin in Lee et al. nov.

Diagnosis: Lunulate scutelliforms up to five lunules; periproct on oral surface; lunules with cross-linked lunule wall.

Remark: This superfamily is proposed here based on the strong molecular support (Fig. 2) and the predicted origination event occurs during the Eocene (Fig. 3).

#### Family Astriclypeidae Stefanini, 1912

Diagnosis: as for superfamily

Distribution: Indo-Pacific West (Fig. 1B)

Remarks: *Astriclypeus*, *Sculpsitechinus* and *Echinodiscus* are included in this study

#### Superfamily Taiwanasteroidea Lin in Lee et al. nov.

Diagnosis: Flattened scutelliforms without lunules.

Remark: Wang^26^ proposed the Superfamily Taiwanasteritida that includes the Family Taiwansteridae and the Family Fibulariidae. Both families are now considered not closely related and this superfamily as defined in Wang^26^ is polyphyletic. Based on cladistic analyses, Wang^50^ proposed the Superfamily Dendrasteracea, including Echinarachniidae, Dendrasteridae and Mellitidae, under the Suborder Scutellina, and Fibulariidae and Laganidae under the Suborder Laganina. Mooi^51^ provided a detailed study on living species of *Dendraster*. During ontogeny, the position of periproct shifts from the margin toward the peristome in all three living *Dendraster* species. While the clade is well defined based on molecular evidence (Fig. 2), it is difficult to define morphologic synapomorphies for this superfamily because *Dendraster* has many autapomorphies. Although *Sinaechinocyamus* was small in size (< 2 cm), it originates in the Taiwan Strait based on the good fossil records in Taiwan since late Miocene^36^. The new clade Taiwanasteroidea proposed here include four extant families: Dendrasteridae, Taiwanasteridae, Scutellidae and Echinarachniidae. This is the only clypeasteroid clade that occurs on both West and East Pacific margins.

#### Superfamily Mellitoidea Lin in Lee et al. nov.

Diagnosis: Lunulate scutelliforms with an anal lunule; with festooned lunule walls^25^ and/or marginal indentations; periproct positioned between peristome and anal lunule.

Remark: This superfamily is proposed here based on the strong molecular support (Fig. 2) and the predicted origination event occurs during the Miocene (Fig. 3).

#### Family Mellitidae Stefanini, 1912

Diagnosis: as for superfamily

Distribution: Their main habitats are along the western margins of North America and South America, and around the Caribbean Sea and Gulf of Mexico^48^.

Remarks: *Lanthonia*, *Leodia*, *Encope*, *Mellitella* and *Mellita* are included in this study.

## Materials and methods

### Ethical approval

No species of echinoderms collected in this study are listed in national laws as protected or endangered. Most of the collected organisms are not subjected to restriction by national or international laws and do not require special permission, except two specimens (see Table 2) collecting in 2017 in Kenting National Park, Taiwan (permit number 1071000145).

**Table 2:** Species and sequences used in the current phylogenetic analysis.

	Family	Species	Locality	Voucher	*cox1*	*16S*	*H3*	*28S*
Camarodonta	Echinometridae	*Colobocentrotus mertensii*	East coast, Taiwan	Inv-10064	**OQ339142**	**OQ308852**		
Spatangoida	Eurypatagidae	*Linopneustes longispinus*			AJ639918	AJ639819		AJ639795
	Maretiidae	*Maretia planulata*	Kenting, Taiwan, 21°56.21′N 120º44.82′E	Inv-10069	**OQ339146**	**OQ308853**		
Cassiduloida	Cassidulidae	*Rhyncholampas pacificus*			MN683981			
	Echinolampadidae	*Conolampas sigsbei*			AJ639902	AJ639800		AJ639777
	Echinolampadidae	*Echinolampas crassa*				DQ073744		
Clypeasteroida	Clypeasteridae	*Arachnoides placenta*	Changhua, Taiwan	Inv-10063	MH837529	**OQ308849**		
		*Clypeaster japonicus*	Sagami Bay, Japan	Inv-10067	MH837530	**OQ308846**		
		*Clypeaster reticulatus*^*a*^	Kenting, Taiwan, 21º56.21′N 120º44.82’E	Inv-10070	**OQ339141**	**OQ308848**		**OQ308839**
		*Clypeaster reticulatus*^*a*^	Green Island, Taiwan	Inv-10073			**OQ341674**	
		*Clypeaster virescens*	Keziliao, Taiwan, 22°43.55′N 120°15.25′E	Inv-10072	**OQ339140**	**OQ308847**	**OQ341673**	
Scutelloida	Echinarachniidae	*Echinarachnius parma*			HM542173			
	Taiwanasteridae	*Sinaechinocyamus mai*	Miaoli, Taiwan	Inv-10065	**OQ339143**	**OQ308842**	**OQ341670**	**OQ308838**
	Astriclypeidae	*Astriclypeus mannii*	Kinmen, Taiwan	Inv-10066	**OQ339144**	**OQ308844**	**OQ341671**	
		*Echinodiscus bisperforatus*^*b*^	Pra Pas Beach, Ranong, Thailand, 9°21.96'N 98°23.67'E	Inv-10071	**OQ339147**	**OQ308845**		
		*Echinodiscus bisperforatus*^*b*^						DQ073763
		*Sculpsitechinus auritus*	Keziliao, Taiwan, 22°43.55′N 120°15.25′E	Inv-10062	**OQ339149**	**OQ308843**	**OQ341669**	
	Dendrasteridae	*Dendraster excentricus*			MK037276			
	Fibulariidae	*Echinocyamus pusillus*			KX458954	DQ073743		DQ073762
	Laganidae	*Laganum fudsiyama*	off Yi-Lan, Taiwan, sta. CP4181, 24°36.279′N 122°27.1804’E, 435 m, R/V ORI, French Beam trawl, KAVALAN 2018 expedition	Inv-10068	**OQ339145**	**OQ308851**	**OQ341672**	
		*Peronella japonica*			LC374903			
		*Peronella lesueuri*	Kinmen, Taiwan	Inv-10061	**OQ339148**	**OQ308850**	**OQ341668**	
	Mellitidae	*Encope aberrans*			MF616973	MF617477		MF617645
		*Encope grandis*			KF204670	KF205050		KF205052
		*Lanthonia longifissa*			MF616955	MF617459		MF617627
		*Leodia sexiesperforata*			MF616941	MF617445		MF617613
		*Mellita notabilis*			KF204742	KF204931		KF205124
		*Mellita isometra*			KF204832	KF205021		KF205214
		*Mellitella stokesii*			MF616945	MF617449		MF617617
	Scutellidae	*Scaphechinus mirabilis*^*c*^	Mutsu Bay, Japan	Inv-10074		**OQ 308841**		
		*Scaphechinus mirabilis*^*c*^			JQ341154;			
		*Scaphechinus mirabilis*^*c*^			AB900169			

^a, b, c^: sequences being combined into single taxon in phylogenetic analyses. Voucher specimens are deposited at the marine invertebrate (inv-) collection of the National Taiwan University Museums (NTUM). New sequences generated in this study are indicated in bold.

### Sample collection and DNA extraction

A total of 12 specimens of echinoids were newly collected and exanimated in this study (Table 2). The habitat of each collected specimen was shown in Table S2. Collected specimens were directly fixed in 95% ethanol prior to DNA extraction. Genomic DNA was extracted from spine muscle by scratching the oral side of the test using the QIAamp DNA Micro-Kit (Qiagen, Hilden). The specimens were kept as vouchers in National Taiwan University Museums (NTUM) (Table 2).

### PCR amplification and sequencing

We performed polymerase chain reactions (PCR) to amplify two mitochondrial genes (cytochrome c oxidase I; *cox1* and 16S ribosomal RNA; *16S*), and two nuclear genes (28S ribosomal RNA; *28S* and Histone H3; *H3*) from each collected specimen (Table 3).

**Table 3:** Primers, annealing temperatures and number of cycles used for PCR and sequencing.

Gene	Primers	Sequences 5′–3′	Source	Annealing	Elongation
*cox1*	EchinoF1	TTT CAA CTA ATC ATA AGG ACA TTG G	Ward et al.^52^	50 °C, 30 s	72 °C, 1 min
	HCO	TAA ACT TCA GGG TGA CCA AAA AAT CA	Folmer et al.^53^		
	COIP2F	GCY ATG AGN GTN ATY ATN CG	Lee et al.^24^	54 °C, 30 s	72 °C, 1 min
	COIP2R	GAG TAT CGY CGN GGC ATT C	Lee et al.^24^		
*16S*	16SarL	CGC CTG TTT AAC AAA AAC AT	Palumbi et al.^54^	46 °C, 30 s	72 °C, 1 min
	16SbrH	CCG GTC TGA ACT CAG ATC ACG T	Palumbi et al.^54^		
*28S*	28SF1	AAC CAG GAT TCC YTY AGT AG	This study	50 °C, 30 s	72 °C, 1 min. 30 s
	28SR1	GAG GGA ACC AGC TAC TAG	This study		
	28S23L	GAC CTC AGA TCG GAC GAG AC	Stockley et al.^55^	52 °C, 30 s	72 °C, 1 min. 30 s
	28S1344R	CAA GGC CTC TAA TCA TTC GCT	Stockley et al.^55^		
*H3*	H3F1	ATG GCT CGT ACC AAG CAG ACV GC	Colgan et al.^56^	53 °C, 30 s	72 °C, 40 s
	H3R1	ATA TCC TTR GGC ATR ATR GTG AC	Colgan et al.^56^		

PCR reactions in a total volume of 25 µl, contained 20–80 ng of DNA template, 0.2 µM of each primer, and 12.5 µl 2 × EmeraldAmp GT PCR Master Mix (TaKaRa Bio.). PCR cycles included an initial denaturation step of 94 °C for 2 min, followed by 35 cycles of denaturation (94 °C, 30 s), annealing and elongation step (Table 3), and a final elongation step at 72 °C for 5 min. The purification and sequencing of PCR products were then carried out at Genomics Company (Taiwan). The obtained DNA sequence chromatograms were assembled using CodonCode Aligner v.6.0.2 (Codoncode Corporation, Dedham, MA, USA). The two overlapping *cox1* sequences (*cox1* part 1 and part 2) were assembled to form a longer *cox1* sequence.

### Phylogenetic analysis

Following the current phylogenetic framework of the Echinoidea^7^, we included 25 species of Luminacea (Cassiduloida, Echinolampadoida, Clypeasteroida, and Scutelloida) and three non-Luminacea species from Spatangoida and Camarodonta in the present phylogenetic analyses. We included *Colobocentrotus mertensii* (Odontophora) as an outgroup to root the inferred phylogenetic trees. The newly obtained *cox1*, *16S*, *28S*, and *H3* sequences together with their orthologous sequences from 17 echinoderm taxa retrieved from Genbank were included in the analyses (Table 2). We aligned the *cox1* and *H3* sequences by eye and *16S* and *28S* sequences by the MAFFT ver. 7 multiple alignment program, applying default parameters^57^. The final trimmed alignments consisted of 1,269 bp, 578 bp, 1,145 bp and 309 bp DNA sequences for *cox1*, *16S*, *28S*, and *H3*, respectively. To gain the power in terms of phylogenetic resolution, we adopted an approach with simultaneous analysis for the multi-gene data. The separated gene datasets with a total of 28 common echinoderm taxa were herein combined for the subsequent analyses. Although some of the individual gene sequences of the included taxa in the concatenated dataset may come from different individuals or be missing at a particular gene locus, we do not expect this to cause a significant error in phylogenetic inference at the interfamilial level.

We performed a phylogenetic analysis using partitioned maximum likelihood (ML) method as implemented in RAxML v.8.0^58^ with the GTR + G substitution model based on the compiled multi-gene dataset. The partitions were set by gene and by codon position for protein coding genes. The parameters including nucleotide substitutions and distribution of site rates in each partition were estimated independently. Thus, the estimated alpha value of the gamma distribution varies among partitions, with values of 0.070, 0.045, and 1.281 for three codon position of *cox1*, respectively, 0.212 for *16S*, 0.063 for *28S*, 0.888, 0.020, and 0.020 for *H3*. Nodal support was assessed by bootstrapping^59^ with 1,000 pseudo-replicates. We also performed a Bayesian Inference (BI) analysis on the combined dataset with MrBayes v.3.2.6^60^ on the CIPRES Science Gateway^61^. Substitution model for each partition was set following the best-fit partition scheme given by PartitionFinder 2^62^ (Table S3). Four Markov chains were performed in each of two parallel runs for 20,000,000 generations, sampling every thousand generation and a heating temperature of 0.02. Convergence was evaluated using Tracer v.1.7^30^ confirming all Effective Sample Size (ESS) values were over 200. The BI tree was summarized using a 50% majority rule tree. Support values from both ML and BI methods were used to evaluate the robustness of inferred phylogenetic relationships within Luminacea.

### Divergence time estimation

Divergence times of lineages were estimated using BEAST v.2.6.7^63^ in a relaxed log-normal clock using the same dataset as for the phylogenetic analyses with eight well-documented echinoderm fossils (see below) for calibration. The Bayesian trees were estimated with a Yule model. We set a single partition for the dataset with GTR + G substitution model. Trees were linked across genes whereas clocks were set unlinked. We followed the previous study of sand dollar phylogeny^48^, by setting the distributions of priors for mean rates (ucld.mean) uniform, ranging from 0.001 to 1 and standard deviation (ucld.stdev) as exponential distribution with a mean equal to 0.3333. Four independent runs of 100 million MCMC generations each were performed and sampled every 1000th generations. Each run was initiated from a random starting time tree. We checked the parameter log files for convergence with Tracer v.1.7. The resulting trees files from the four independent runs were removed 10% of trees as burn-in in each run, and combined using LogCombiner v.2.6.7. The maximum clade credibility (MCC) tree with mean divergence times generated from BEAST was reconstructed using TreeAnnotator v.2.6.4.

Our phylogenetic tree was time-calibrated using echinoid fossils that provide hard minimum and soft maximum bounds through exponential distributions in which the 95% credibility interval was equal to the maximum age of the strata where the first known fossil excavated, or the maximum bound of the most recent common ancestor (MRCA) that has been estimated in previous studies. The fossils and references used in the analysis were shown in Table S1.

### Ancestral area reconstruction

The origin and patterns of geographical diversification of Luminacea taxa were assessed by ancestral area reconstruction using the Dispersal-Extinction-Cladogenesis (DEC) model implemented in RASP v. 4.2^31^ with the time-calibrated consensus tree reconstructed using BEAST (see above). Terminal taxa were regarded as the representatives for their genera (Fig. 3). We defined eleven biogeographical units for the included genera according to previous studies^15,18^ with minor modifications based on oceanic basin, continental shelf, surface currents, and regional endemicity^18^ as follows: (a) Tropical eastern Indian and western Pacific Ocean (EIWP); b) Southern Australia and New Zealand (SANZ); (c) Northwestern Pacific (NWP); (d) Tropical western Indian Ocean (WIO); e) Northeastern Pacific (NEP); (f) Northwestern Atlantic (NWA); (g) tropical eastern Pacific (EP); h) tropical western Atlantic and Caribbean Sea (WA); (i) Northeastern Atlantic and Mediterranean (NEA); j) Tropical eastern Atlantic (EA); k) South Africa (SAFR) (Fig. 4A).

### Biogeography network visualization

For further geographic visualization of biogeography we built StrainHub networks from the phylogenetic data and metadata using the strainhub R package^39^. The network of connections between geographic provinces is based on the tree used in RASP analysis and the source/hub ratio (SHR). The size of the circles represents the SHR of the nodes in the network (Fig. 4B) with larger, circles indicating the relative importance of a geographic province as a source of lineages. The thickness of the lines indicates the frequency of movement of lineages from one body of water to another.

### Equipment and settings

Images of sand dollars in Figs. 3 and S3 were taken by J.-P.L. using a Nikon D750 digital SLR camera fitted with a macro lens (AF-S VR Micro-Nikkor 105 mm f/2.8G IF-ED) and were edited in Adobe Photoshop CS6. Brightness and contrast were adjusted with the software ImageJ.

### Supplementary Information

Supplementary Information.

## Data Availability

New data generated and or analyzed during the current study are available in GenBank under the Accession numbers OQ339140—OQ339149 for *cox1*, OQ308841—OQ308853 for *16S*, OQ341668—OQ341674 for *H3* and OQ308838—OQ308839 for *28S*.
