# Supplementary material for: Phylogeny, ancestral ranges and reclassification of sand dollars
Source: Sci Rep. 2023 Jun 23;13:10199. doi: 10.1038/s41598-023-36848-0 (PMC10290142; doi:10.1038/s41598-023-36848-0)
Supplement: Supplementary file 1 — Supplementary Information. [file 41598_2023_36848_MOESM1_ESM.pdf]

Phylogeny, ancestral ranges and reclassification of sand dollars

Hsin Lee[1,2,3+], Kwen-Shen Lee[4,+], Chia-Hsin Hsu[2], Chen-Wei Lee[2], Ching-En Li[2], Jia-Kang Wang[2], Chien-Chia Tseng[2], Wei-Jen Chen[3], Ching-Chang Horng[2], Colby T. Ford[5,6,7,8], Andreas Kroh[9], Omri Bronstein[10,11], Hayate Tanaka[12], Tatsuo Oji[13], Jih-Pai Lin[2,*], Daniel Janies[7,8]

[1]National Museum of Marine Biology and Aquarium, Pingtung, 944401, Taiwan

[2]Department of Geosciences, National Taiwan University, Taipei, 10617, Taiwan

[3]Institute of Oceanography, National Taiwan University, Taipei, 10617, Taiwan

[4]Biology Department, National Museum of Natural Science, Taichung, 40453, Taiwan

[5]Tuple LLC, 2413 Commonwealth Ave, Charlotte, North Carolina, 28205, USA

[6]School of Data Science, University of North Carolina at Charlotte, 9201 University City Blvd, Charlotte, North Carolina, 28223 USA

[7]Department of Bioinformatics and Genomics, University of North Carolina at Charlotte, 9201 University City Blvd, Charlotte, North Carolina, 28223 USA

[8]Center for Computational Intelligence to Predict Health and Environmental Risks (CIPHER), University of North Carolina at Charlotte, 9201 University City Blvd, Charlotte, North Carolina, 28223 USA

[9]Department of Geology and Palaeontology, Natural History Museum Vienna, Vienna, 1010, Austria

[10]School of Zoology, Faculty of Life Sciences, Tel Aviv University, Tel Aviv, 6997801, Israel

[11]Steinhardt Museum of Natural History, Tel Aviv University, Tel Aviv, 6997801, Israel

[12]Department of Biological Sciences, Graduate School of Science, University of Tokyo, Tokyo, 113-0033, Japan

[13]University Museum, Nagoya University, Furo-cho, Nagoya, 464-8601, Japan

***Correspondence and requests for materials should be addressed to J.-P.L. (email: alexjplin@ntu.edu.tw; xyloplax1@gmail.com)

+These authors contributed equally to this work

Electronic Supplementary Material

Previous classifications of Clypeasteroida discussed

Mortensen 1948 (ref.[1])

Order Clypeasteroida
- Suborder Clypeastrina
  - Family Clypeastridae
  - Family Arachnoididae
- Suborder Laganina
  - Family Fibulariidae
  - Family Laganidae
  - Family Scutellidae

Durham 1955 (ref.[2])

Order Clypeasteroida
- Suborder Clypeasterina
  - Family Clypeasteridae
  - Family Arachnoididae
- Suborder Laganina
  - Family Fibulariidae
  - Family Laganidae
  - †Family Neolaganidae
- Suborder Scutellina
  - Family Scutellidae
  - †Family Protoscutellidae
  - †Family Eoscutellidae
  - Family Dendrasteridae
  - Family Echinarachniidae
  - †Family Monophorasteridae
  - Family Mellitidae
  - Family Astriclypeidae
  - †Family Abertellidae
  - †Family Scutasteridae
- Suborder Rotulina
  - Family Rotulidae

Wang 1984 (ref.[3])

Order Clypeasteroida

Suborder Clypeasterina
Family Clypeasteridae
Family Arachnoididae
Suborder Scutellina
Infraorder Scutellidea
Family Scutellidae
Family Astriclypeidae
Infraorder Laganidea
Superfamily Taiwanasteritida
Family Taiwansteridae
Family Fibulariidae
Superfamily Laganitida
Family Laganidae
†Family Neolaganidae
Superfamily Rotulitida
Family Rotulidae

Wang 1992 (ref.[4])
Order Oligopygoida
†Family Oligopygidae
†Family Protolampadidae
Order Clypeasteroida
Suborder Lenitina
†Family Lenitidae
Suborder Laganina
Family Fibulariidae
†Family Sismondiidae
Family laganidae
†Family Neolaganidae
†Family Proescutellidae
†Family Tarphypygidae
Family Rotulidae
Suborder Clypeasterina
Family Clypeasteridae
Family Arachnoididae
†Family Fossulasteridae
Suborder Scutellina
†Family Eoscutidae
†Family Protoscutellidae
Family Scutellidae
Family Astriclypeidae
Infraorder Kewiidea

†Family Kewiidae
Superfamily Dendrasteracea
Family Echinarachniidae
Family Dendrasteridae
Family Mellitidae
Superfamily Eoscutellacea
†Family Eoscutellidae
†Family Abertellidae
†Family Monophorasteridae
†Family Scutasteridae

Schultz 2005 (ref.[5])
Order Clypeasteroida
Suborder Clypeasterina
Family Clypeasteridae
Family Arachnoididae
†Family Fossulasteridae
†Family Scutellinoididae
Suborder Laganina
Family Fibulariidae
Family Laganidae
†Family Neolaganidae
Family Rotulidae
Suborder Scutellina
†Family Scutellinidae
†Family Protoscutellidae
†Family Eoscutellidae
†Family Scutellidae
Family Echinarachniidae
†Family Abertellidae
Family Dendrasteridae
†Family Scutasteridae
Family Astriclypeidae
†Family Monophorasteridae
Family Mellitidae

Schultz 2017 (ref.[6])
Clypeasteroida
Clypeasterina
Arachnoididae

Clypeasteridae
Scutellina
Scutelliformes
Taiwanasteridae
Echinarachniidae
Scutellidae
Echinarachniidae
Dendrasteridae
Astriclypeidae
Mellitidae
Laganiformes
Fibulariidae
Laganidae
Rotulidae

Kroh 2020 (ref.[7])
Stem group Neognathostomata
†Family Clypeidae
†Family Nucleolitidae
Crown group Neognathostomata
Family Apatopygidae
†Family Pygaulidae
†Family Archiaciidae
†Family Clypeolampadidae
Order Clypeasteroida
Stem group Clypeasteroida
†Family Faujasiidae
†Family Plesiolampadidae
†Family Conoclypeidae
†Family Oligopygidae
Crown group Clypeasteroida
Family Clypeasteridae
†Family Fossulasterinae
Echinolampadacea
Order Cassiduloida
Suborder Cassidulina
Family Cassidulidae
Suborder Neolampadina
Family Neolampadidae
Order Echinolampadoida
Family Echinolampadidae

Order Scutelloida
- Stem group Scutelloida
  - †Family Scutellinidae
- Crown group Scutelloida
  - Infraorder Laganiformes
    - Family Fibulariidae
    - Family Laganidae
  - Infraorder Scutelliformes
      - Family Taiwanasteridae
    - Stem group Scutelliformes
      - †Family Protoscutellidae
    - Crown group Scutelliformes
      - Family Echinarachniidae
      - Family Dendrasteridae
      - Family Rotulidae
      - †Family Scutellidae
      - †Family Eoscutellidae
      - †Family Scutasteridae
      - †Family Abertellidae
      - Family Astriclypeidae
      - †Family Monophorasteridae
      - Family Mellitidae

Mongiardino Koch et al. 2022 (ref.[8])

Irregularia
- Atelostomata
  - Holasteroida
  - Spatangoida
- Neognathostomata
  - Luminacea
    - Clypeasteroida
    - Echinolampadacea
      - Cassiduloida
      - Scutelloida
        - Scutelliformes

RASP DEC Analysis result

DEC result file of Name
[TAXON]
1 Rhyncholampas_pacificus G
2 Conolampas_sigsbei AH
3 Echinolampas_crassa AHJ
4 Ech18_Arachnoides_placenta A
5 Clypeaster_reticulatus ABCDGHJK
6 Ech106_Clypeaster_virenscens ABCDGHJK
7 Ech32_Clypeaster_japonica ABCDGHJK
8 Echinocyamus_pusillus ACDHIJ
9 Ech43_Laganum_fudsiyama ABCD
10 Ech13_Peronella_lesueuri ABCD
11 Peronella_japonica ABCD
12 Ech30_Astriclypeus_manni AC
13 Ech14_Sculpsitechinus_auritus ABD
14 Ech49_Echinodiscus_bisperforatus ACD
15 Ech29_Sinaechinocyamus_mai AC
16 Dendraster_excentricus EG
17 Echinarachnius_parma CEF
18 Scaphechinus_mirabilis C
19 Lanthonia_longifissa G
20 Mellita_isometra H
21 Mellita_notabilis G
22 Mellitella_stokesii G
23 Leodia_sexiesperforata FH
24 Encope_aberrans H
25 Encope_grandis G
[TREE]
Tree=(((1:106.214225,(2:6.490878,3:6.490878):99.723346):8.551437,((4:39.028993,(5:18.015538,(6:4.489178,7:4.489178):13.52636):21.013455):64.088521,(8:52.439209,(9:35.070895,(10:18.000753,11:18.000753):17.070141):17.368315):50.678305):11.648147):5.138475,((12:50.388548,(13:39.354713,14:39.354713):11.033834):40.337605,((15:32.872547,(16:26.781921,(17:20.996038,18:20.996038):5.785883):6.090626):10.672663,((19:15.7555,(20:5.410499,21:5.410499):10.345001):3.852497,(22:17.564041,(23:15.345302,(24:12.062763,25:12.062763):3.282539):2.218739):2.043956):23.937213):47.180942):29.177984);
[RESULT]
DEC results:
node 26 (LR): AHJ 74.23 AH 25.77
node 27 (LR): A 67.25 G 22.65 H 10.10
node 28 (LR): A 41.04 C 6.35 G 6.27 D 6.19 B 6.16 H 5.83 J 5.76 E 5.61 F 5.60 I 5.59 K 5.59
node 29 (LR): A 100.00

node 30 (LR): A 100.00
node 31 (LR): ABCD 27.17 A 6.82 ACD 6.69 ABC 6.69 ABD 6.64 AC 5.55 AD 5.51 AB 5.50 BCD 5.14 C 4.14 D 4.07 B 4.06 CD 4.03 BC 4.02 BD 3.98
node 32 (LR): ABCD 22.17 A 13.84 ACD 6.28 ABC 6.27 ABD 6.19 AC 6.16 AD 6.08 AB 6.06 C 4.74 D 4.52 B 4.48 BCD 3.83 CD 3.17 BC 3.15 BD 3.08
node 33 (LR): A 38.38 ABCD 14.15 C 9.41 D 8.73 B 8.60 AC 7.00 AD 6.88 AB 6.86
node 34 (LR): A 100.00
node 35 (LR): A 86.31 G 13.69
node 36 (LR): A 69.69 D 16.18 AD 14.13
node 37 (LR): A 68.76 AC 16.82 C 14.42
node 38 (LR): C 75.91 CE 24.09
node 39 (LR): C 28.04 CE 25.20 CG 24.77 CEG 13.58 E 8.40
node 40 (LR): C 42.27 CG 22.11 CE 12.95 AC 8.55 CEG 7.59 ACEG 6.52
node 41 (LR): GH 100.00
node 42 (LR): G 100.00
node 43 (LR): GH 100.00
node 44 (LR): GH 100.00
node 45 (LR): G 63.99 GH 36.01
node 46 (LR): G 68.29 GH 31.71
node 47 (LR): CG 31.02 G 17.28 CEG 10.06 ACEG 9.53 ACG 9.17 CGH 7.20 C 6.26 AG 5.20 CEFG 4.30
node 48 (LR): A 32.87 C 26.35 AG 10.00 AC 9.92 G 9.76 ACG 5.60 CG 5.50
node 49 (LR): A 3.26 ACG 2.24 AG 2.24 AC 2.18 ACDG 1.80 ACGH 1.73 ACEG 1.64 ABCG 1.57 ACGJ 1.49 ACFG 1.37 ADG 1.31 ACD 1.30 ACH 1.28 ACGI 1.28 ACGK 1.28 AGH 1.26 ABG 1.15 AEG 1.14 ABC 1.14 ACE 1.11 AH 1.10 ACJ 1.09 AGJ 1.09 AD 1.09 ACDH 1.03 ADGH 1.01 AFG 0.98 ABCD 0.98 ACF 0.97 ABDG 0.95 AE 0.95 AB 0.94 AGK 0.92 ACI 0.91 ACK 0.91 ADEG 0.91 ACDE 0.90 AJ 0.90 ABCH 0.90 ABGH 0.89 ADGJ 0.88 ACEH 0.88 AGHJ 0.88 AEGH 0.88 ACDJ 0.88 ACHJ 0.86 ABEG 0.80 AF 0.80 ACDF 0.79 ADFG 0.79 ABCE 0.78 ABGJ 0.78 ACFH 0.77 ABCJ 0.77 AFGH 0.76 AEGJ 0.76 ACDI 0.75 ACDK 0.75 ADGI 0.75 ADGK 0.75 AK 0.74 AI 0.74 ACEJ 0.74 ACHI 0.72 ACHK 0.72 AGHI 0.72 AGHK 0.72 ADH 0.71 AEFG 0.70 ABFG 0.69 ABCF 0.68 ACEF 0.68 AFGJ 0.66 ABGI 0.65 ABGK 0.65 AEGK 0.65 AEGI 0.65 ACFJ 0.65 ABCI 0.65 ABCK 0.65 ABD 0.64 ACEI 0.63 ACEK 0.63 AGJK 0.62 ABH 0.62 ADE 0.62 ACIJ 0.61 ACJK 0.61 AEH 0.61 ADJ 0.60 AHJ 0.59 CG 0.58 AFGK 0.56 AFGI 0.56 ACFI 0.55 ACFK 0.55 ABE 0.54 ADF 0.53 AFH 0.52 ABDH 0.52 AGIK 0.52 ABJ 0.52 ACIK 0.52 AEJ 0.51 ADK 0.50 ADI 0.50 ADEH 0.50 ADHJ 0.50 AHK 0.49 CDG 0.49 AEF 0.46 ABF 0.46 ABDE 0.45 CGH 0.45 ABDJ 0.44

Supplementary tables

Table S1. Fossils and references used in the divergence time analysis.

Taxa	Earliest fossil / Reference	Time interval	Ma	Locality
Split between outgroups and Luminacea	*Clypeus*† sp.[9]	Middle Jurassic	170.3–168.3	Dhruma Formation (Shaqra Group), Saudi Arabia
Cassiduloida + Echinolampadoida	Most recent common ancestor estimated in ref [10]	Early Cretaceous	146.6–102.5	
Astriclypeidae	*Echinodiscus tiliensis*†[11]<br>*Echinodiscus yeliuensis*†[11]	Late Paleocene to Early Eocene	58.7–48.6	Tili Village, Nantou, Taiwan<br>Yeliu, Taiwan
Clypeasteridae	*Clypeaster profundus*†[12]	Late Eocene	41.3–38.0	Turkey
Laganidae	*Peronella archerensis*†[13]	Late Eocene	38.0–33.9	Inglis Limestone Formation, Florida
Mellitidae	*Mellita* sp.[14]	Early Miocene	20.44–15.97	Baitoa Formation, Dominican Republic
Encope	*Encope* sp.[15]	Middle Miocene	16–11.6	Lowest Gatun Formation, Sand Dollar Hill, Panama
Mellita	*Mellita aclinensis*†[16]	Early Pliocene	5.3–3.6	Tamiami Formation, Florida

Table S2. Habitat of sand dollars collected in this study

Species	habitat
Colobocentrotus mertensii	Intertidal or subtidal zone, rocky shore
Maretia planulata	Subtidal zone, sandy bottom
Arachnoides placenta	Intertidal to subtidal zone, sandy bottom
Clypeaster japonicus	Subtidal zone, sandy bottom
Clypeaster reticulatus	Subtidal zone, sandy bottom, with a depths range shallower than *C. virescens*
Clypeaster virescens	Subtidal zone, sandy bottom, with a depths range deeper than *C. reticulatus*
Sinaechinocyamus mai	Intertidal to subtidal zone, sandy bottom
Astriclypeus mannii	Intertidal to subtidal zone, sandy bottom
Echinodiscus bisperforatus	Intertidal to subtidal zone, sandy bottom
Sculpsitechinus auritus	Intertidal to subtidal zone, sandy bottom
Laganum fudsiyama	Shallow water to deep sea (50–654 m) sandy bottom[17]
Peronella lesueuri	Intertidal to subtidal zone, sandy bottom
Scaphechinus mirabilis	Intertidal to subtidal zone, sandy bottom

Table S3. Best-fit substitution models obtained from PartitionFinder 2. The suffix after each gene name denotes the codon position. The models of DNA substitution are: SYM (Zharkikh, 1994)[18], HKY (Hasegawa et al., 1985)[18], GTR (Rodríguez et al., 1990)[18], F81 (Felsenstein, 1981)[18], and JC (Jukes and Cantor, 1969)[18].

**Partition**	cox1_1	cox1_2	cox1_3	16S	28S	H3_1	H3_2	H3_3
**Best model**	SYM+I+G	HKY+I	GTR+I+G	GTR+I+G	GTR+I+G	GTR	F81	JC+I

Supplementary figures

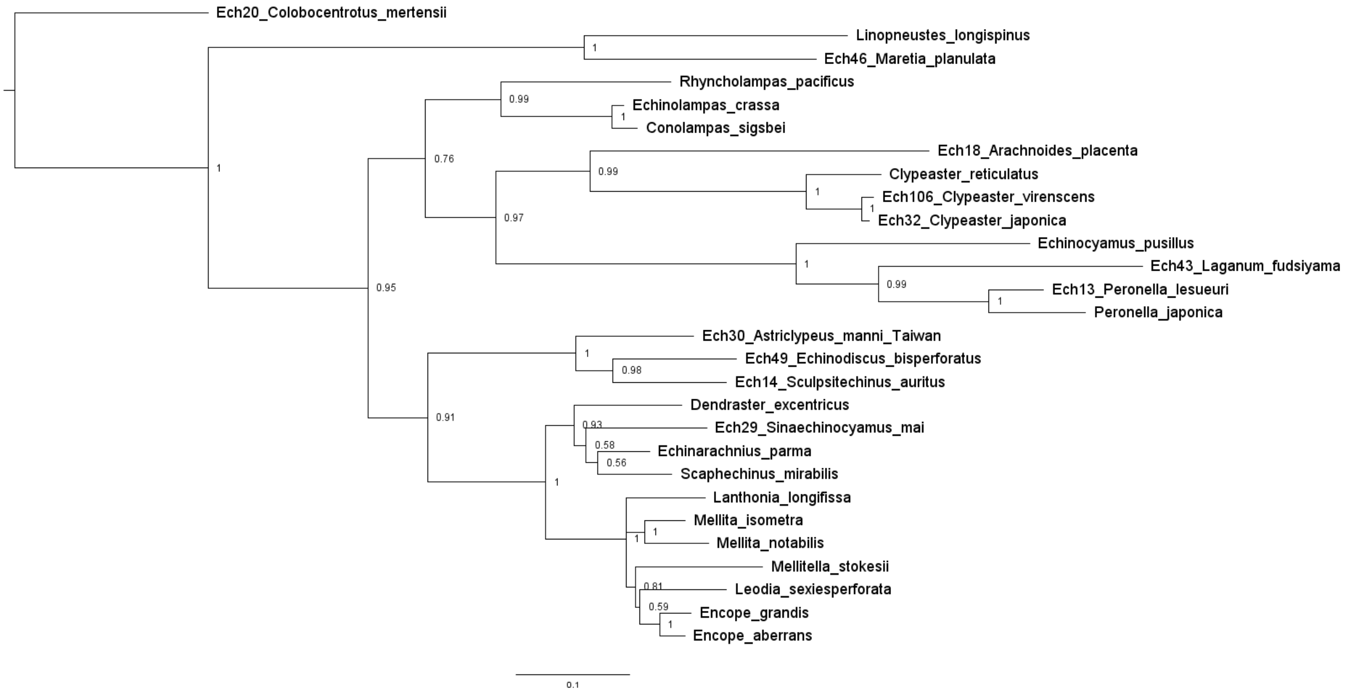

Fig. S1. Phylogenetic relationships of Neognathostomata based on Bayesian Inference analysis.

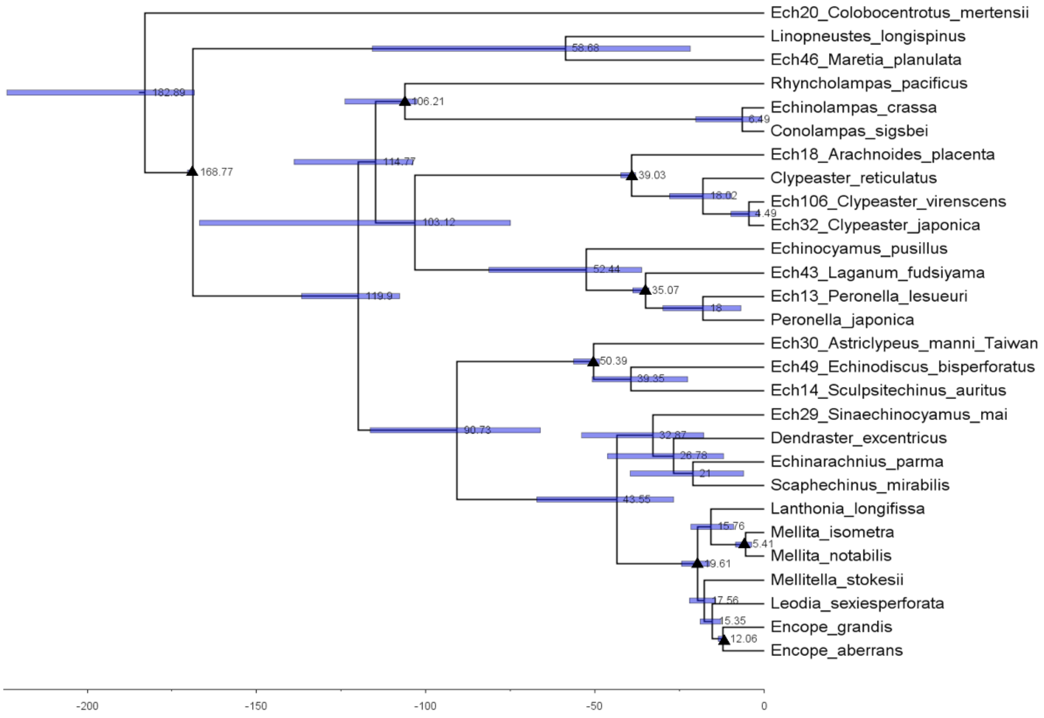

Fig. S2. Rates of evolution for studied echinoids with error bars representing 95% confidence Level (see Fig. 3 and related text). Nodes with black triangles represent the constrained and assigned age priors.

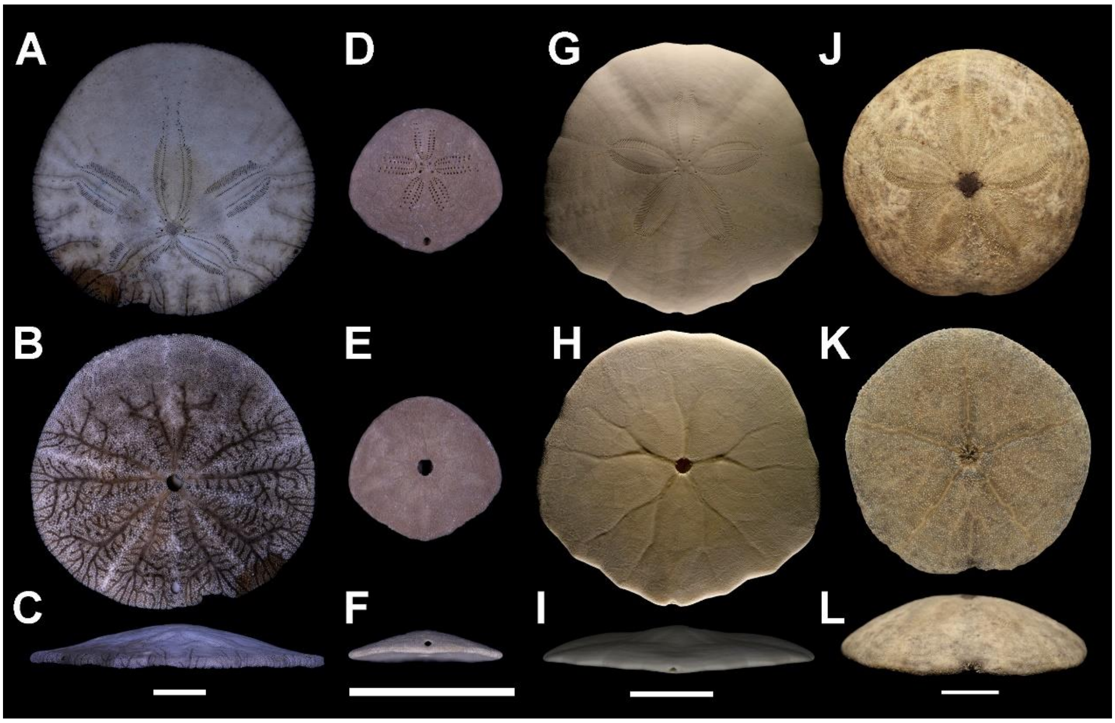

Fig. S3. Key non-lunulate scutelliformes included in this study. All scales = 10 mm. A-C. *Dendraster excentricus* (Eschscholtz, 1831). D-F. *Sinaechinocyamus mai* (Wang, 1984). G-I. *Scaphechinus mirabilis* A. Agassiz, 1864. J-L. *Echinarachnius parma* (Lamarck, 1816). Image credit: Jih-Pai Lin (A-C; G-I) and Kwen-Shen Lee (D-F; J-L).

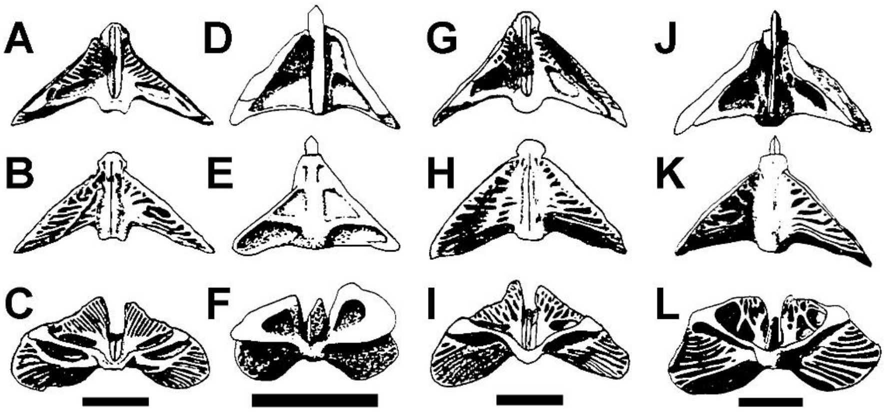

Fig. S4. Views of pyramid morphology of key non-lunulate scutelliformes discussed in this study (modified from Wang[4]). Scales = 0.5 mm (D-F); 3 mm (A-C; G-L). A-C. *Dendraster excentricus*; D-F. *Sinaechinocyamus mai*; G-I. *Scaphechinus mirabilis*; J-L. *Echinarachnius parma*.

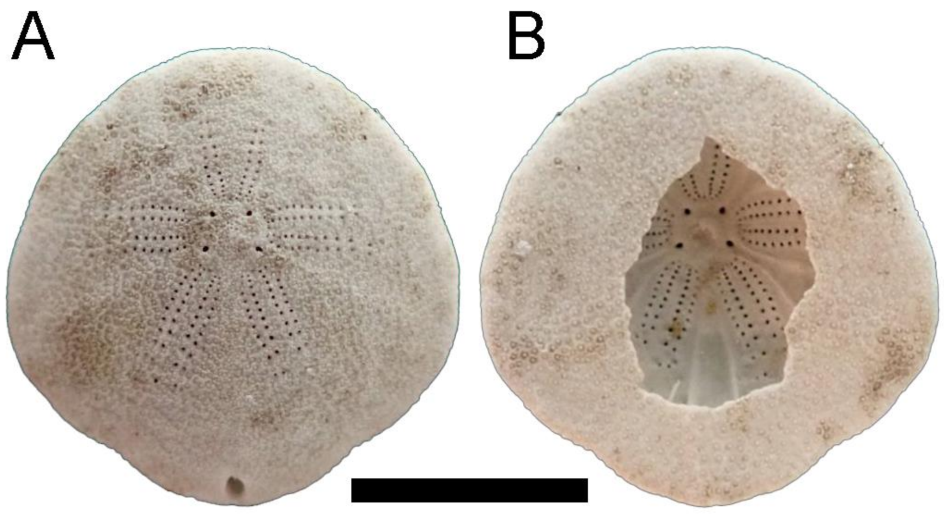

Fig. S5. New occurrence of *Sinaechinocyamus* sp. from Seto Inland Sea, Japan. Denuded test. Scale = 5mm. Image credit: Hayate Tanaka.

References

1 Mortensen, T. *A Monograph of the Echinoidea IV. 2. Clypeasteroida. Clypeasteridæ, Arachnoidæ, Fibulariidæ, Laganidæ and Scutellidæ. Text*. 1-471 (C. A. Reitzel, 1948).

2 Durham, J. W. Classification of clypeasteroid echinoids. *University of California Publications in Geological Sciences* **31**, 73-198 (1955).

3 Wang, C.-C. New classification of clypeasteroid echinoids. *Proceedings of the Geological Society of China* **27**, 119-152 (1984).

4 Wang, C.-C. *Phylogenetic analysis of the clypeasteroid echinoids* PhD thesis thesis, Yale University, (1992).

5 Schultz, H. *Sea Urchins: A guide to worldwide shallow water species*. 1-484 (Heinke & Peter Schultz Partner Scientific Publications, 2005).

6 Schultz, H. *Echinoidea, Volume 2: Echinoidea with Bilateral Symmetry. Irregularia*. 1-359 (Walter de Gruyter GmbH, 2017).

7 Kroh, A. in *Sea Urchins: Biology and Ecology, 4th Edition* Vol. 43 (ed J. M. Lawrence) 1-17 (Academic Press, 2020).

8 Mongiardino Koch, N. *et al.* Phylogenomic analyses of echinoid diversitification promt a re-evaluation of their fossil reocord. *eLife* **11**, e72460 (2022).

9 Manivit, J., Le Nindre, Y.-M. & Vaslet, D. Le Jurassique D`Arabie Centrale. *Document du BRGM* **4**, 25-519 (1990).

10 Mongiardino Koch, N. & Thompson, J. R. A total-evidence dated phylogeny of Echinoidea combining phylogenomic and paleontological data. *Systematic Biology* **70**, 421-439 (2021).

11 Wang, C.-C. Fossil Echinodiscus from Taiwan. *Bulletin of the central geological survey* **3**, 107-115 (1984).

12 Pinar, N. Sur les oursins de l'Eocène moyen de Catalca-Karacaköy (Trakkya, Turquie). *Bulletin de la Société Géologique de France* **1**, 35-53 (1951).

13 Puri, H. S. & Vernon, R. O. *Summary of the geology of Florida and a guidebook to the classic exposures*. (Florida Geological Survey Special Publication 5, 1964).

14 Saunders, J. B., Jung, P. & Biju-Duval, B. Neogene Paleontology in the Northern Dominican Republic: 1. Field Surveys, Lithology, Environment, and Age. *Bulletins of American Paleontology* **89**, 1-79 (1986).

15 Coppard, S. E. & Lessios, H. A. Phylogeography of the sand dollar genus *Encope*: implications regarding the Central American Isthmus and rates of molecular evolution. *Scientific Reports* **7**, 11520, doi:10.1038/s41598-017-11875-w (2017).

16 Oyen, C. W. & Portell, R. W. Diversity patterns and biostratigraphy of Cenozoic echinodems from Florida. *Palaeogeography, Palaeoclimatology, Palaeoecology* **166**, 193-218 (2001).

17 Lane, D. J. W., Marsh, L. M., VandenSpiegel, D. & Rowe, F. W. E. Echinoderm fauna of the South China Sea: an inventory and analysis of distribution patterns. *The Raffles Bulletin of Zoology* **Suppl. 8**, 459–493 (2000).

18 Posada, D., & Crandall, K. A. MODELTEST: testing the model of DNA substitution. *Bioinformatics* **14**, 817–818 (1998).
